# Systematic review of prospective studies assessing risk factors to predict anorexia nervosa onset

**DOI:** 10.1186/s40337-023-00882-0

**Published:** 2023-09-20

**Authors:** Jean-Philippe Charrat, Catherine Massoubre, Natacha Germain, Aurélia Gay, Bogdan Galusca

**Affiliations:** 1 TAPE (Eating Disorders, Addictions and Extreme Bodyweight) Laboratory, University Jean Monnet, Saint Etienne, France; 2 Centre TCA, Hôpital Nord, Batiment A, CHU Saint Etienne, 42055 Saint Etienne Cedex 2, France; 3 Referral Center for Eating Disorders, Saint Etienne University Hospital, Saint Etienne, France; 4 Addictology Department, Saint Etienne University Hospital, Saint Etienne, France

**Keywords:** Systematic review, Anorexia nervosa, Prospective studies, Risk factors, Predictors

## Abstract

**Background:**

According to case‒control studies, a multitude of factors contribute to the emergence of anorexia nervosa (AN). The present systematic review examines prospective studies specifically designed to evaluate the prediction of AN onset.

**Methods:**

According to the ARMSTAR 2 and PRISMA 2020 checklists, the PubMed, PsycINFO and Cochrane databases were searched. The methodological quality of the studies was assessed with the Downs and Black checklist.

**Results:**

Three articles concerning prospective studies of the general population were ultimately included in the review. The methodological quality of these studies was not optimal. Bidirectional amplification effects were observed between risk factors, some of which could have a relative predictive force as low bodyweight or body dissatisfaction. Even if not included according to specified criteria for this systematic review 11 longitudinal studies, with retrospective analysis of AN onset’ prediction, were also discussed. None of these studies asserted the predictive value of particular risk factors as low body weight, anxiety disorders or childhood aggression.

**Conclusions:**

To date there are insufficient established data to propose predictive markers of AN onset for predictive actions in pre-adolescent or adolescent populations. Future work should further evaluate potential risk factors previously identified in case‒control/retrospective studies within larger prospective investigations in preadolescent populations. It is important to clearly distinguish predisposing factors from precipitating factors in subjects at risk of developing AN.

**Supplementary Information:**

The online version contains supplementary material available at 10.1186/s40337-023-00882-0.

## Abstract (plain language summary)

Currently health care guidelines for eating disorders do not include proposal of markers to predict the onset of anorexia nervosa. The current work provides a systematic review of the scientific literature concerning this subject. To date only three published studies were designed in a prospective longitudinal way to evaluate potential predictors of anorexia nervosa onset. When taking into account these studies only low bodyweight and body dissatisfaction in early puberty were proposed as predicting elements for further anorexia nervosa development. Meanwhile the prediction precision was calculated for none of them. No other psychological elements were retained or studied. The age range of the population entering the follow up in these studies was too large covering the peak age of anorexia nervosa itself (13–16 years of age). Larger prospective studies including prepubescent individual and evaluating more psychological markers (perfectionism, negative affectivity or negative self-evaluation) or environmental ones are needed.

**Supplementary Information:**

The online version contains supplementary material available at 10.1186/s40337-023-00882-0.

## Background

Anorexia nervosa (AN), bulimia nervosa (BN), and binge eating disorder (BED) are the most common eating disorders (EDs) [1]. AN is a severe psychiatric disorder whose etiology remains unknown [2]. The lifetime prevalence of AN is estimated to be 0.3–0.9 for women [3–8]. The incidence of AN according to DSM-5 criteria is 25.7 per 100,000 persons per year [9]. It occurs most often in the peripubertal period. Incidence rates for AN are significantly higher for females aged 15–19 years, accounting for approximately 40% of all identified cases [10, 11]. The average point prevalence rate is approximately 0.29% in young females [12]. AN is a psychiatric disorder that is associated with significant morbidity, and several recent studies have confirmed its high mortality rate [13–16]. Given these circumstances It is important to identify early, during the peripubertal period, risk factors capable of predicting the onset of anorexia nervosa and thus to establish effective preventive actions.

The results of several case‒control studies show a typical psychometric profile of subjects at risk of AN. In particular, some studies have demonstrated the importance of perfectionism, negative affectivity and negative self-evaluation as factors of personal vulnerability to develop AN [17–21]. These results converge with those of a study of twins [22]. Studies based on the Oxford Risk Factors Interview (RFI) [23] demonstrated a significantly greater level of exposure to several personal vulnerability factors in subjects with anorexia compared to control cases [17, 18, 21, 22]: perfectionism, negative affectivity, negative self-evaluation, extreme compliance, the absence of close friends, and a history of family depression.

Some environmental factors are common in other psychiatric disorders. These include maternal and paternal parenting difficulties, family discord, and parental mood disorders [21]. Subjects with AN have an increased sensitivity to more than half of the environmental factors included in the RFI [17, 18, 21, 22]. The contribution of family history of diet problems, weight problems or eating disorders does not seem to be significant [17, 21, 22]. It should be noted that a history of sexual abuse is clearly associated with an increased risk of a lifetime eating disorder diagnosis [24, 25].

The role of proximal environmental events precipitating AN pathology is not clearly established. Less than half of subjects with AN reveal experiencing a significant life event in the year before disease onset, and only a quarter indicates experiencing a severely negative life event [26, 27]. According to other studies, particularly serious events (e.g., the loss of a first-degree relative) seem to play a role as proximal triggers for the onset of AN [28]. However, the overall rate of negative events reported for these subjects is not higher than that for matched community controls, although young persons who develop AN tend to report more extreme negative events that primarily affect the family sphere.

Although several potential risk factors have been pointed out by case‒control studies, very few longitudinal population studies have evaluated risk factors for the onset of AN.

Therefore, the aim of the current work was to perform a systematic review of prospective studies performed to evaluate the prediction the onset of AN.

### Aim of the review

This study was a systematic review conducted to identify and evaluate original articles written in English and outline the results of prospective studies on the association between risk factors/predictors and the onset of AN. The aim was to answer the following questions: What are the reliable potential predictors of the development of AN, identified by prospective studies among the multiple risk factors (psychological, familial, other) to which subjects are exposed in initially non-syndromic populations? Ultimately, what is the accuracy/reliability of the predictors identified by prospective studies in distinguishing subjects who eventually develop AN from those who do not?

Case control studies and retrospective analysis within longitudinal cohorts, not designed for the study of AN onset’ prediction, were not included into the review design. Case controls bring important knowledge on potential predictors associated with AN onset but they are not designed to evaluate the prediction consistency, reliability etc. Retrospective studies found associations and correlations and suggest potential predictive factors; these factors should be tested in prospective studies. The advantage of the prospective study is that it follows a fixed protocol for its entire duration. It allows the number of new cases to be calculated directly over a given period (incidence rate). The prospective study would evaluate the reliability of a presumed predictive factor. However, additionally to the retained articles within the present systematic review, longitudinal studies with retrospective analysis of AN onset prediction were also discussed.

### Search strategy

The research protocol was developed in cooperation with a medical team of the ED department (psychiatrists and endocrinologists). The research protocol was established with reference to the critical appraisal tool ARMSTAR 2 [29, 30]. This guideline leads the researcher through a succession of sixteen questions to which the answers are: yes, no, partial yes. The manuscript was written considering the PRISMA 2020 checklist [31], which is a 27-item checklist that helps a searcher develop the approach for identifying studies and structure the writing process.

**Fig. 1:**
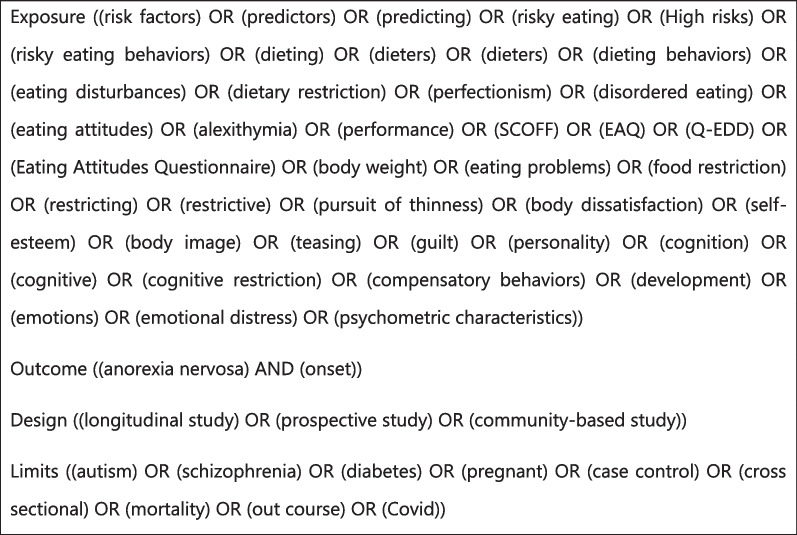
Search strategy for the PubMed, PsycINFO and Cochrane databases

The research question was established using the PICO method (Population, Intervention, Comparator, Outcome). After several preliminary tests and according to our prior knowledge of the subject, key words were included to more broadly identify potential studies that used expressions synonymous to those of prospective studies (e.g., longitudinal studies). Finally, to see all potential studies investigating certain aspects of anorexia, new terms were included (e.g., alexithymia). A saturation threshold was obtained when the addition of new terms in the equation did not modify the numerical results obtained. All of these steps were developed and validated by consensus of the authors (Fig. 1).

The following databases were investigated: the PubMed, PsycINFO and Cochrane databases. Preliminary research using the databases showed that no systematic review has been previously carried out on the same subject. The research protocol was registered on the International Prospective Register of Systematic Reviews, PROSPERO (ID CRD42022295971). The published protocol is accessible with the following link: https://www.crd.york.ac.uk/PROSPEROFILES/295971_PROTOCOL_20220418.pdf.

To supplement the database searches, all the references of the selected articles were reviewed. Moreover, gray literature was searched for, but no article matching the main criteria was found.

### Inclusion and exclusion criteria

The inclusion criteria were as follows: original studies published in a journal with an editorial board and peer review. Prospective studies specifically designed to research and analyze risk factors/predictors specific to AN were included. The subjects of the studies were recruited from the general female population and were not from diagnosed clinical populations at first evaluation. The subjects were identified as not being affected by an eating disorder at the start of the study. The subjects of the studies included female adolescents or preadolescents at first evaluation. Moreover, the duration of the reported studies was greater than 12 months.

Studies including diagnosed clinical populations at the first evaluation point were excluded.

As mentioned above case control studies and retrospective analysis into longitudinal cohorts not specifically designed to evaluate the prediction of AN onset were not included in the main analysis.

Subjects who had developed clinical or subclinical forms of AN at the end of the study were compared with those who did not develop AN.

### Outcomes

#### Main outcome

The main outcome was the accuracy (including measures of specificity, sensitivity, and ROC curves) of psychological or somatic markers in predicting the onset of clinical or subclinical AN. The disorder was identified using the current DSM diagnostic criteria, and the outcome was tested at the end and over the duration of the prospective study.

#### Additional outcomes

The relationship between potential risk factors and the onset of clinical and subclinical AN was measured at the end and over the duration of the prospective study.

### Selection and extraction of the articles

Two of the authors of the systematic review used the following three standard steps to select the studies independently: analyzing the articles’ titles, reading the titles and abstracts, and reading the full texts.

Data were extracted independently. Only articles that met all the established criteria were included. After selection of the studies, the relevant data were recorded in a standardized spreadsheet (Excel). The data were extracted and summarized according to the following standard aspects: main author, publication date, sample size, subject age, duration of the study, duration of the follow-up and control periods, tools and methods used in each control period, proportion of subjects lost to follow-up at the last control period, subpopulations and the criteria used to define them, main outcomes (AN threshold, AN subthreshold, eventually AN traits), the prevalence of the different clinical forms at the end of the study, the results of the review and assessment of risk factors, the statistical methods used, the assessment of the reliability of each predictive marker (Yes or No), and the methodology used for the assessment of the reliability of the predictive markers.

The researchers were blinded to each other’s decisions. At each selection and extraction step, if there were divergences, a third author was consulted. A fourth expert from the eating disorders referential center could also be called upon to assist in the decision-making process. A consensus or majority determined the final conclusion. Finally, all cumulative data were verified once more by the three principal investigators.

### Evaluation of study methodological quality

To assess the quality and possible biases of the articles selected, the validated checklist for nonrandomized studies, preestablished by Downs and Black [32], was used. This checklist is based on twenty-seven items that assess the risk of bias in five areas: study quality, external validity, study biases, confounding and selection biases, and study power.

With reference to previously published systematic reviews, this checklist was adapted for observational studies. Items 8, 13, 14, 15, 23 and 24 were eliminated. Item 27 was scored if the statistical power of the survey was specified in the article [33]. In keeping with the design of our review, Item 4 was eliminated because it did not study the effects of treatments or therapeutic methods. All items received a score of 0 or 1 point, except Item 5, which received a score of 0, 1 or 2 points. The adjusted scale received a total score ranging from 0 to 21 points.

### Search summary and analysis tables

**Fig. 2:**
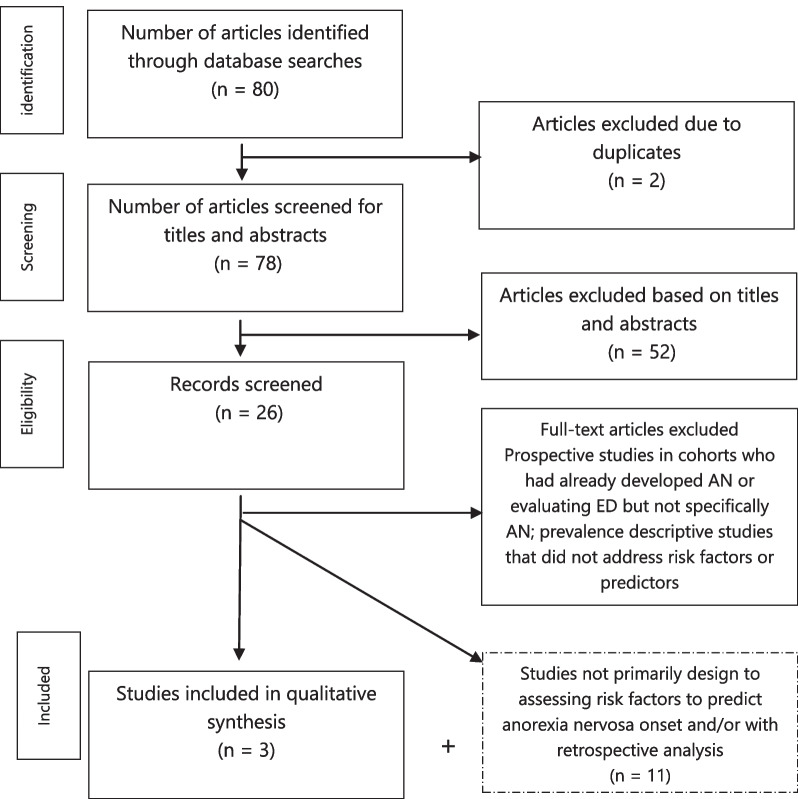
Study selection flow diagram for the current systematic literature review according to the PRISMA statement

The search resulted in 80 articles (72 from PubMed; 4 from PsycINFO; 4 from Cochrane) (Fig. 2). After excluding duplicates, 78 titles and abstracts were examined; 26 full texts were selected for reading. 52 articles were excluded after reading the abstract for the following reasons: prospective studies of cohorts who had already developed AN, prospective studies evaluating risk factors for the onset of ED but not specifically AN and prevalence descriptive studies that did not address risk factors or predictors.

After reading the full texts, 3 studies were formally selected. The following data concerning the selected studies are presented in Table 1: authors, year of publication, country, cohort size, age, follow-up period, measuring instruments, statistical method, retained risk factors and outcome measurement, defined as the development or absence of a disorder. The results are presented in alphabetical order using the last names of the first authors. Finally, the tables include the dependent variable (consequence studied = the development of a disorder), the main results and the control variables used in the analyses.

**Table 1:** Summary of the populations, design characteristics and main results of the selected studies

Authors	Year of pub	Country	Cohort size	Initial age	Follow-up period	Measuring risks factors tolls	ED Diagnoses tools	Results					Attrition bias
								Statistical method	Retained risk factors	Association with AN onset		Cut-off calculation	
Stice et al. [45]	2017	USA	257	12–16 years	Follow up at 2 and 8 years	Ideal-Body Stereotype Scale, Thinness expectancies scale, 5-item scale about denial of the cost of pursuing thin ideals, Weight Control Scale, Dutch Restrained Eating Scale, Negative Affect Scale-Revised, Studies-Depression Scale, Beck Depression inventory, Functional impairment measured with 17 items from the Social Adjustment Scale-Self Report for Youth, Frequency of visits to mental health care institutions, BMI	EDDI, DSM-5 criteria for eating disorders	Univariate, multivariate Cox analysis		Subthreshold/Threshold AN		No	Not taken into account
										HR	p value		
									Functional impairment Body Mass Index	1.71 0.05	.0047 < .0001		
										Threshold AN			
										HR	p-value		
									Negative affect Functional impairment Body Mass Index	2.06 2.17 0.16	.0144 .0292 .0201		
Stice and Desjardins [46]	2018	USA	1272	Mean age 18 years	3 years	8-item Ideal-Body Stereotype Scale–Revised, 9-item Thinness Expectancy scale, 5 items scale about denial of cost of pursuing thin ideal, 9 items from the Satisfaction and Dissatisfaction with Body Parts Scale, 5-item Weight Control scale, Dutch Restrained Eating Scale, Negative Affect Scale Revised, Center for Epidemiologic Studies-Depression Scale, Beck Depression Inventory, 17 items from the Social Adjustment Scale-Self Report for Youth, Frequency of visits to mental health care, BMI	EDDI, DSM-5 criteria for eating disorders	Multivariate analysis, Classification tree	Low BMI	Participants in top 83% of BMI: Incidence = 0.3%	Part. in bottom 17% BMI: Incidence = 11.1%	No	Not taken into account
									Interaction of low BMI/high body dissatisfaction	Part. in bottom 17% BMI and bottom 89% of body dissatisfaction: Incidence = 9.0%	Part. in bottom 17% BMI and top 11% of body dissatisfaction: Incidence = 71.4%		
										Overall classification rate = 0.982 Null tree classification rate = 0.979 F-index = 0.303 G-mean = 0.438			
Tyrka et al [50]	2002	USA	1272	Mean age 18 years	3 years	SCID, EAT-26, SIQYA, Family Environment Scale	EDI, EAT 26, Structured Clinical Interview for DSM-III-R	Multivariate analysis, Logistic regression	Perfectionism BMI	T2	T3	No	Not taken into account
										Unstandardized B Coeff. = .746; -.319 p-value ≤ .05	Unstandardized B Coeff = 1.314; -.817 p-value = ≤ .05		

Among the full text articles selected for reading 12 (listed in Additional file 1) were excluded for the same reasons as above. For the rest of 11 articles, it was particularly difficult for the research team to agree on. Their design is prospective but the approach to identifying risk factors in these articles is clearly retrospective [34–43]. Although these are prospective studies, they were not initially or specifically designed to predict the development of AN [34–44]. In addition, for some of the articles the diagnosis of AN was not made by a clinician but was composed from questionnaire data [34, 36–41, 43]. Even if not formally included into the study these studies were summarized in Table 3.

## Main text

The three selected studies had different sample characteristics. They were all conducted in the USA and included only women. Two studies from the same author used the same sample of 1272 subjects with an average age of 18 years and a 3-year follow-up [45, 46]. The sample consisted of combined cohorts previously related by an efficacy trial [47] and two effectiveness trials [48, 49]. The rate of loss to follow-up at the end of these studies was 7% at 6 months and 14% at 3 years. The third study used a sample of 257 participants aged between 12 and 16 years, with a follow-up of 8 years [50]. The rate of loss to follow-up at the end of this study was 15%.

The articles used different statistical methods: multivariate [50], univariate and multivariate with Cox analysis [45], and classification tree methods [46]. It should be noted that the tools were less detailed in one article [50]. The evaluation criteria thresholds were poorly specified in one article [45] and not specified at all in the other [46]. Finally, there was no comparative approach between a population that was at risk and a population that was not at risk in any of the three articles. At the end of the follow-up period, the prevalence of AN was in line with that described in cross-sectional studies.

Quality assessment according to the Downs and Black checklist showed overall scores between 12 and 14 (Table 2). According to the Downs and Black scale, the mean of all three articles is within the fair quality level. In the external validity domain, no article was clear about how the participants were selected, and two articles did not specify the sample representability. In the internal validity domain, no article used data dredging. Only two articles used an accurate method (valid and reliable) to measure the outcomes. In the confounding domain, no article described characteristics of the participants who were lost to follow-up between the initial selection process and the final sample, and no article performed control for attrition bias in the final analysis. Finally, no study reported a power calculation for the sample size, and they were all classified as having a “risk of bias” in the power domain (Table 2). In addition to the Downs and Black analysis, it was necessary to add other possible biases that were not included in the main checklist: retrospective reports for lifetime diagnosis history, a small number of cases, a high prevalence of a lifetime eating disorder history [50], AN and BN patients included at baseline, and an average age at baseline that was too high given the state of knowledge concerning the onset age of eating disorders [45, 46]. It could be considered a bias that recruitment was performed within a large age range (from secondary school to college) [45, 46], thus covering both peaks of AN incidence [3]. Within this cohort, people who had already developed AN were naturally excluded from the analysis, leading to a diminished risk for AN onset within the remaining study population [45, 46].

**Table 2:** Summary of quality assessment with the Down and Black Checklist

	Stice et al. [45]	Stice and Desjardins [46]	Tyrka [50]
*Reporting*			
Aims are clear	1	1	1
Main outcomes are clear	1	1	1
Characteristics of the patients are clear	0^a^	1	1
Confounders in each group clear	1	1	1
Main findings are clear	1	1	1
Estimates of the random variability	1	1	1
Losses to follow-up	0	0	1
*P value*s reported	1	1	1
*External validity*			
Representative selection process	0^b^	0b	0
Representative sample	0	1	0
*Internal validity*			
Data dredging	1	1	1
Different lengths of follow-up	1	1	1
Statistical tests	1	1	1
Accurate main exposure measurement	0	1	0
Accurate outcome measurement	1	0	1
*Confounding*			
All participants from the same population	1	1	1
Participants selected simultaneously	1	1	1
Adjustment for confounding	0	0	0
Losses	0	0	0
*Power*			
Sample power calculation	0	0	0
Score	12^c^ (16^d^)	14 (19)	14 (19)

^a^The cohorts were derived from three previously published prevention trials. The cohorts are described in the articles reporting the results of these three trials, but are not described in this article, which focused on predictors of eating disorders.

^b^In the article, no characteristic mentioned by the author made it possible to confirm the representativeness of the sample, although in the prevention trials from which the cohort was drawn, the author affirmed sample representativeness in terms of distribution by ethnicity and parental education level.

^c^Score according to the adapted B & D scale: maximum 21.

^d^Adjusted score according to the original B & D scale: maximum 28.

Two of the articles focused on the importance of negative affect and/or negative emotions in the development of the pathology [45, 50]. The impact of perfectionism was evaluated in one article [50]. Psychosocial characteristics, disease onset in high school and family functioning were not identified as predictors. A low BMI was considered important in one article and was considered independent of other factors [45]. The importance of “being pretty” was determined to be the main factor in one article [50]. Moreover, an amplified interaction was related to high body dissatisfaction and low BMI in one article [46]. Ultimately, no article was able to state that any risk factor is indeed predictive of AN with a sufficient degree of certainty. There is a multitude of risk factors, some of which are close to having a predictive capacity, such as a low BMI. Above all, what seemed to be retained in these results is the interaction effect among different risk factors whose concomitant presence is potentially predictive, generating a specific morbidity profile for the subjects.

## Discussion

In the scope of this systematic review, a total of only three prospective studies were identified, specifically tailored to investigate and analyze risk factors and predictors unique to Anorexia Nervosa (AN). This limited inclusion of studies might potentially exert an impact on the comprehensive nature of the review's findings. These studies collectively suggest that among the potential predictors of AN onset, prepubescent low BMI and body dissatisfaction emerge as noteworthy candidates. However, it is crucial to underscore that the precision of their predictive capacity was either suboptimal or, in some cases, not even calculated.

Upon scrutiny, the methodological aspects of the selected studies were determined to be generally fair. Nevertheless, a prevalent bias observed was rooted in the insufficient comparison between populations considered to be at risk and those that were not, alongside the somewhat ambiguous delineation of these populations. It is also important to note all the studies were conducted in the USA. This geographical limitation raises pertinent concerns about the extent to which the outcomes could be extrapolated internationally. The relationship among body image, disordered eating, and sociocultural factors seems to differ across different countries [51]. The complex interplay of cultural factors may have potentially influenced the composition of the study cohorts, their baseline definitions, and, consequently, the conclusions drawn from the data extracted.

Concerning two papers [45, 46], the sample was completed in the context of evaluating an intervention for eating disorder prevention [47–49]. These studies explicitly acknowledged the possible impact of their sampling procedures on the results and went on to assert that the effects of risk factors remained statistically intact despite the interventions. [45]. These studies included only young women at high risk for eating disorders due to body dissatisfaction. Thus, no direct comparison with a control group (no body dissatisfaction) was performed in terms of further AN incidence. At first sight, the results could not be generalized to other population. Indeed, examining individuals with a particular risk factor can impact results in favor of risk factors correlated with that status [46]. However, the high-risk for AN sample was determined with a panel of factors not based solely on body dissatisfaction but also elevated thin-ideal internalization and dieting [46]. The authors note that the means and standard deviations of the risk factors were very close to those obtained in a community sample of age-matched adolescents. This pleads in favor of the representativeness of the sample. In addition, the authors rely on previous community studies that found similar risk factors in the general adolescent population [52–56]. This last point tends to confirm that the results are not biased by the high-risk design. However, the confirmatory factor analyses measures did not exhibit unidimensionality, so the authors were unable to perform formal tests of factorial invariance across samples. So, by considering all these precisions, the authors approach can be considered as constituting a debatable source of partial methodological bias.

Scrutinizing the methodologies applied by Stice et al. [45], 46] unveiled certain definitional concerns. Relying exclusively on a binary response to a question concerning body image concerns could inadvertently encompass individuals who are otherwise healthy. This challenge of accurately demarcating a high-risk population solely based on body dissatisfaction highlights the complexity of this endeavor. It's important to recognize that a high-risk population may embody a spectrum of psychometric characteristics, each with varying degrees of severity [17–22]. This holistic perspective, embracing psychological dimensions beyond body dissatisfaction, is crucial in avoiding a myopic understanding of risk factors.

In the process of excluding individuals with eating or consumptive disorders (cancer, sepsis, malabsorption, etc.), the cohort comprising individuals with a low BMI could potentially encompass those characterized by constitutional thinness (CT), a natural state with no signs of undernutrition [57], particular genetics [58] and a resistance to weight gain [59]. This population is often misdiagnosed as having AN [60]. Importantly, given their metabolic profile, individuals with constitutional thinness might exhibit more pronounced weight loss in the context of restrictive eating behaviors compared to the general population. Interestingly, in their study, Stice and Desjardins [46] found that low BMI was the most potent predictor of AN onset. This assertion prompts a consideration of whether this predictor might particularly correspond to cases with overt somatic manifestations. This viewpoint indicates that the prediction of AN onset might not be uniformly applicable across all presentations, potentially excluding subthreshold forms with comparatively less conspicuous low body weight expressions [61, 62].

AN presents with two incidence peaks at 14 and 18 years of age [3]. Consequently, prospective studies intended to assess predictors of AN onset should ideally focus on subjects younger than 13 years of age, spanning the early secondary school phase. Critically, the three studies under review encompassed a wide age range, inadvertently incorporating both of these incidence peaks. The challenge posed by the low incidence of AN extends further to the conundrum of precisely defining thresholds and subthresholds that can effectively guide research endeavors. Central to this issue is the definition of populations at risk, a question demanding methodological rigor. A plausible approach entails deriving hypotheses by amalgamating findings from case–control studies—a strategy founded on the identification of risk factors from populations characterized by AN. However, the crux lies in the need to subject these risk factors to rigorous testing across a diverse spectrum of subjects, commencing as early as the preadolescent stage, to ascertain their true predictive potential.

This literature review could be perceived as excessively restrictive due to the rigorous selection criteria employed. The review specifically concentrated on prospective studies that explicitly assessed the predictive value in AN onset, thereby excluding longitudinal studies with retrospective evaluations of risk factors, even if they showcased temporal associations with AN development. These latter studies, although not formally incorporated into the review, undoubtedly provide insights into the relationships between various precipitating factors and AN onset (see Table 3). Their data highlighted the association between childhood traumatic factors [34, 36, 42] and AN onset, the link between anxiety and AN onset [35, 38, 63], low BMI [40], drive for thinness [37], early childhood temperament and psychopathology [43], obsessive compulsive disorders or depression [44] impact on AN symptoms and traits onset. However, these investigations were not primarily designed to quantitatively measure the accuracy of their predictive potential in the context of AN onset. It's imperative to underscore that these studies would not have fundamentally altered the overarching conclusions drawn from the systematic review.

**Table 3:** Other studies looking for predictors of anorexia nervosa but not initially designated for this purpose

Authors	Year of pub	Country	Cohort size	Initial age	Follow-up period (years)	Initial cohort objective	Measuring risks factors tolls	ED Diagnoses tools	Results				Attrition bias
									Statistical method	Retained risk factors	Association with AN onset	Cut-off calculation	
Sanci L et al.[34]	2008	Australia	1936	16 to 24	11	Investigation of mental health, personality, behaviors, school, family, drug and alcohol use	CSA (childhood sexual aggression) before the age of 16 years (retrospectively ascertained at the age of 24 years)	Branched Eating Disorders Test, 9-item Adolescent Dieting Scale	Cross-sectional odds ratios, discrete-time proportional hazards models	CSA without physical contact and with physical contact	CSA without physical contact: 1 report: OR = 1.2 (0.19–7.7); ≥ 2 reports: OR = 2.4 (0.8–7.2) CSA with physical contact: 1 report: OR = 1.1 (0.17–7.5); ≥ 2 reports: OR = 1.6 (0.46–5.6)	No	Multiple imputation
Buckner JD et al.[44]	2010	USA	1709	16	14	Epidemiology of depression	K-SADS, K-SADS-P	Structured clinical interview DSM-IV, non-patient version	Multivariate logistic regression, odds ratios	Obsessive–compulsive disorder (OCD)	OCD: OR = 49,26, 95% CI: 1.57 to 1541.47, p = .03	No	Taken into account
Meier SM et al.[35]	2015	USA	1709	16	14	Epidemiology of depression	K-SADS	SCID-for DSM-IV non-patient version	Multivariate logistic regression, odds ratios	Specific anxiety disorders	OCD: OR = 49.26, p = 0.03	No	Take into account
Copeland WE et al.[36]	2015	USA	1420	9 to 16	10	Child psychiatric epidemiology	Parent report and self-questionnaire on childhood bullying involvement	CAPA/YAPA eating disorder module assesses DSM-III-R and IV symptoms	Regression models with robust Variance, OR, MR	Bullying victims Bullies	Any AN symptom: OR = 4.6, 95% CI: 1.8–11.4, p = 0.001 OR = 1.9, 95% CI: 1.2–3.2, p = 0.01 Total AN symptom: no significant result	No	No
Peñas-Lledó E et al.[37]	2016	Sweden	615	19 to 20	3	Investigation of Child and Adolescent Development	Psychometric questionnaires	Binary auto test response	K-means clustering	Drive for thinness and/or negative affect scores	Drive for Thinness-Anxiety/Depression cluster had the highest prevalence of AN at follow-up compared with all other clusters	No	No
Ranta K et al. [38]	2017	Finland	3278	15 to 16	2	Adolescents’ mental health study	Self-reported SPIN, Self-reported Beck Depression Inventory	Self-reported questionnaires’ DSM IV criteria	Chi-square statistics, OR	Depression	OR = 3.6 1.3–10.1), p = 0.015	No	Taken into account
Schaumberg K et al.[39]	2019	England	7767	10	6	Global health study cohort	Parental symptoms Psychometric scores, BMI	Questionnaire using DSM-5 criteria, Parental report of AN symptoms at ages 14 and 16	EFA, Geomin rotation, ESEM	Anxiety symptoms	OR = 1.10; p = 0.01	No	Taken into account
Yilmaz Z et al. [40]	2019	England	1502	12	5	Subslamp from a global health study cohort	BMI	Self-reported ED disorders based on the DSM 5 criteria, self-reported ED behaviors, parental reports of fear of fatness and restricting behaviors	Parametric and non-parametric analyses	BMI at age of 12	Girls: lower BMIs by 4 years (B = − 0.505, SE 0.22) Boys: lower BMIs by 2 years (B = − 0.87, SE 0.38)	No	Take into account
Lloyd EC et al.[41]	2020	England	Study 1: 14,882 Study 2: several cohorts[66, 68, 69]	10	Max 14	2 studies combined: global health study cohort + Mendelian randomization	Psychometric scores, parental report	Parental report of AN symptoms at ages 14 and 16. And Self child report at 14, 16, 18, 24	Multiple logistics regression, multivariate, OR, Mendelian randomization	Anxiety symptoms	Study 1: Worry at age 10: OR = 1.60, 95% CI: 0.93 to 2.77, p = 0.09; anxiety disorder criteria at 10: OR = 2.85, 95% CI: 1.22 to 6.63, p = 0.02 Study 2: Worry increases AN risk: OR = 1.79, 95% CI: 1.25 to 2.55, p = 0.001	No	Not clear
Talmon A et Spatz Widom C [42]	2021	USA	807	0 to 11	Max 41	Abused and/or neglected children followed into adulthood	Documented childhood maltreatment, psychometric scores	Diagnostic Interview Schedule, Version 4	Logistic and linear regression, OR, adjusted OR	Neglect Physical abuse Sexual abuse	OR: 1.32, p = 0.10 OR: 1.10, p = 0.02 OR: 1.53, P = 0.01	No	Taken into account
Bufferd SJ et al.[43]	2022	USA	609	3 to 6	Max 12	Risk factors for psychopathology	Psychometric questionnaires	DSM-IV anorexia nervosa (AN) and bulimia nervosa (BN) symptoms	Multivariate linear regression model	Perceptual sensitivity, Oppositional Defiant Disorder (ODD) and depressive disorder in early childhood	r correlation index from 0.11 to 0.15 (p < 0.05)	No	Take into account

To establish a future prospective study on the predictive risk factors for anorexia, several lessons can be extracted from this literature review.

A particular study design can be proposed. The study should follow a population with a homogeneous age including individuals who are early preadolescent at the beginning of the study. A large cohort needs to be retained and cooperation with school health services is the most appropriate method to conduct this study. It would probably be useful to train physicians and school nurses in the latest knowledge about eating disorders. In doing so, the aim would be to achieve an optimal level of competence in their participation to the study. The study should be conducted over a long period of time in order to cover both age peaks of anorexia nervosa incidence. Thus, it is suitable to establish the study period over the four years of middle school and three years of high school. However, the prolonged duration of such a study could inadvertently trigger attrition. Individuals identified with AN diagnosis at the beginning of the study may not be included in the cohort. Ethical considerations underscore the importance of ensuring that all the individuals diagnosed with ED during the study are provided with appropriate care.

Risk factors to evaluate should not be limited to body dissatisfaction or low BMI. More testing of data from case‒control studies should be evaluated, such as perfectionism, negative affectivity, negative self-evaluation, extreme compliance and other psychometric characteristics, psychiatric and somatic comorbidities. Similarly, environmental factors need to be further tested including: family discord and parental mood disorders [17, 18, 21, 22], the interaction of the environment with the psychometric and biometric characteristics [64] or the potential role of the triggering effect of life events such as the loss of a first-degree relative [28]. The strength of associations among potential predictive factors, as discerned from case–control studies, is far from uniform. In this context, a meta-analysis or systematic review could emerge as a powerful tool to discern the degree of association potency across the spectrum of potential predictors. This approach facilitates an ordered classification of these factors based on their potential relevance.

At last, the understudied domain of biological and neurobiological factors beckons for exploration. The literature presents compelling evidence supporting the relevance of these factors in AN etiology [65–67]. Additionally, the intricate interaction between biological underpinnings and sociocultural factors, such as the pursuit of the thin ideal, resonates with their involvement in the reward systems of the brain [68]. So, it would be relevant to include biological and neurobiological risk factors in future studies. However, it's acknowledged that practical constraints might complicate the inclusion of these factors. Genetic elements have been also identified to define a particular risk profile for the development of AN [69–73]. However, these studies were driven in AN patient’s cohorts. Further prospective studies are needed to set the real link between some characterized polymorphisms preadolescent population and AN onset.

The systematic review, upon comprehensive analysis, underscores the prominence of prepubescent low BMI and body dissatisfaction as conceivable predictors of AN onset. However, it is important to recognize the provisional nature of these findings, as the precision and reliability of their predictive value remain equivocal. In light of the extant data, it is incumbent to exercise caution in extrapolating these findings to practical applications such as prevention programs or policy formulation. A more comprehensive exploration of a diverse range of risk factors, spanning psychological, environmental, and biological dimensions, within a prospective study paradigm is imperative. Only through this nuanced approach can a robust and reliable toolkit be developed for the prediction of AN onset. Such a toolkit, rooted in a multidimensional understanding of the disorder's genesis, holds the key to the implementation of effective prevention initiatives.

### Supplementary Information

**Additional file 1**. Addendum 1.

## Data Availability

Data sharing is not applicable to this article as no datasets were generated or analyzed during the current study.
