# Supplementary material for: Systematic review of prospective studies assessing risk factors to predict anorexia nervosa onset
Source: J Eat Disord. 2023 Sep 20;11:163. doi: 10.1186/s40337-023-00882-0 (PMC10510169; doi:10.1186/s40337-023-00882-0)
Supplement: Supplementary file 1 — Additional file 1. Addendum 1. [file 40337_2023_882_MOESM1_ESM.docx]

Addendum 1. 12 studies excluded after full text reading according to following criteria: prospective studies in cohorts who had already developed AN or evaluating ED but not specifically AN; prevalence descriptive studies that did not address risk factors or predictors

1. Bulik CM, Sullivan PF, Tozzi F, et al (2006) Prevalence, heritability, and prospective risk factors for anorexia nervosa. Arch Gen Psychiatry 63:305–312. https://doi.org/10.1001/archpsyc.63.3.305
2. Herle M, Stavola BD, Hübel C, et al (2020) A longitudinal study of eating behaviours in childhood and later eating disorder behaviours and diagnoses. Br J Psychiatry 216:113–119. https://doi.org/10.1192/bjp.2019.174
3. Johnson-Sabine E, Wood K, Patton G, et al (1988) Abnormal eating attitudes in London schoolgirls--a prospective epidemiological study: factors associated with abnormal response on screening questionnaires. Psychol Med 18:615–622. https://doi.org/10.1017/s0033291700008291
4. Killen JD, Taylor CB, Hayward C, et al (1996) Weight concerns influence the development of eating disorders: a 4-year prospective study. J Consult Clin Psychol 64:936–940. https://doi.org/10.1037//0022-006x.64.5.936
5. Kotler LA, Cohen P, Davies M, et al (2001) Longitudinal relationships between childhood, adolescent, and adult eating disorders. J Am Acad Child Adolesc Psychiatry 40:1434–1440. https://doi.org/10.1097/00004583-200112000-00014
6. Liechty JM, Lee M-J (2013) Longitudinal predictors of dieting and disordered eating among young adults in the U.S. Int J Eat Disord 46:790–800. https://doi.org/10.1002/eat.22174
7. McKnight Investigators (2003) Risk factors for the onset of eating disorders in adolescent girls: results of the McKnight longitudinal risk factor study. Am J Psychiatry 160:248–254. https://doi.org/10.1176/ajp.160.2.248
8. Patton GC, Selzer R, Coffey C, et al (1999) Onset of adolescent eating disorders: population based cohort study over 3 years. BMJ 318:765–768. https://doi.org/10.1136/bmj.318.7186.765
9. Silén Y, Sipilä PN, Raevuori A, et al (2020) DSM-5 eating disorders among adolescents and young adults in Finland: A public health concern. Int J Eat Disord 53:520–531. https://doi.org/10.1002/eat.23236
10. Stice E, Van Ryzin MJ (2019) A prospective test of the temporal sequencing of risk factor emergence in the dual pathway model of eating disorders. J Abnorm Psychol 128:119–128. https://doi.org/10.1037/abn0000400
11. Stice E, Marti CN, Rohde P (2013) Prevalence, incidence, impairment, and course of the proposed DSM-5 eating disorder diagnoses in an 8-year prospective community study of young women. Journal of Abnormal Psychology 122:445–457. https://doi.org/10.1037/a0030679
12. Striegel-Moore RH, Silberstein LR, Frensch P, Rodin J (1989) A prospective study of disordered eating among college students. International Journal of Eating Disorders 8:499–509. https://doi.org/10.1002/1098-108X(198909)8:5<499::AID-EAT2260080502>3.0.CO;2-A
